# Temporal survival trends in cutaneous squamous cell carcinoma accounting for competing risks: A long-term cohort study in Japan

**DOI:** 10.1016/j.jdin.2026.09.017

**Published:** 2026-10-09

**Authors:** Akihiko Yuki, Sumiko Takatsuka, Tatsuya Takenouchi

**Affiliations:** Department of Dermatology, Niigata Cancer Center Hospital, Niigata, Japan

**Keywords:** aging, competing risks, cutaneous squamous cell carcinoma, disease-specific mortality, epidemiology, Japan

*To the Editor:* Cutaneous squamous cell carcinoma (cSCC) is one of the most common skin cancers and represents an increasing clinical burden worldwide.^1^ Because older patients have a substantial risk of dying from causes unrelated to cSCC, conventional survival estimates may be difficult to interpret. Kaplan-Meier methods censor competing deaths and can overestimate the cumulative probability of disease-specific events.^2^ We analyzed a 30-year Japanese cSCC cohort to evaluate temporal survival trends while accounting for competing mortality.

This retrospective cohort study included patients with histologically confirmed cSCC diagnosed at Niigata Cancer Center Hospital between 1991 and 2020. Patients were categorized into early (1991-2000), middle (2001-2010), and late (2011-2020) periods. The primary outcome was cSCC-specific death, with deaths from other causes treated as competing events. Overall survival was estimated using Kaplan-Meier analysis. Cumulative incidence functions were compared using the Gray test, and adjusted analyses were performed using Fine-Gray regression. Detailed methods are provided in the Supplementary Material, available via Mendeley at https://data.mendeley.com/datasets/b5g4zc43z2/1.

A total of 1004 patients were included. Patient characteristics are summarized in Supplementary Table 1, available via Mendeley at https://data.mendeley.com/datasets/b5g4zc43z2/1. Overall survival declined over time (Fig 1), with 5-year survival rates of 75.3%, 65.0%, and 58.7% in the early, middle, and late periods, respectively (log-rank *P* < .001). In contrast, the 5-year cumulative incidence of cSCC-specific death was 6.4%, 6.7%, and 2.3%, respectively (Gray test, *P* = .001; Fig 2), whereas the cumulative incidence of other-cause death increased across study periods (Gray test, *P* < .001; Supplementary Fig 1, available via Mendeley at https://data.mendeley.com/datasets/b5g4zc43z2/1). After adjustment for age, sex, tumor site, and stage, diagnosis during the late period remained independently associated with a lower risk of cSCC-specific death than diagnosis during the early period (subdistribution hazard ratio, 0.41; 95% CI, 0.17-0.98; *P* = .04; Supplementary Table 2, available via Mendeley at https://data.mendeley.com/datasets/b5g4zc43z2/1).

**Fig 1:**
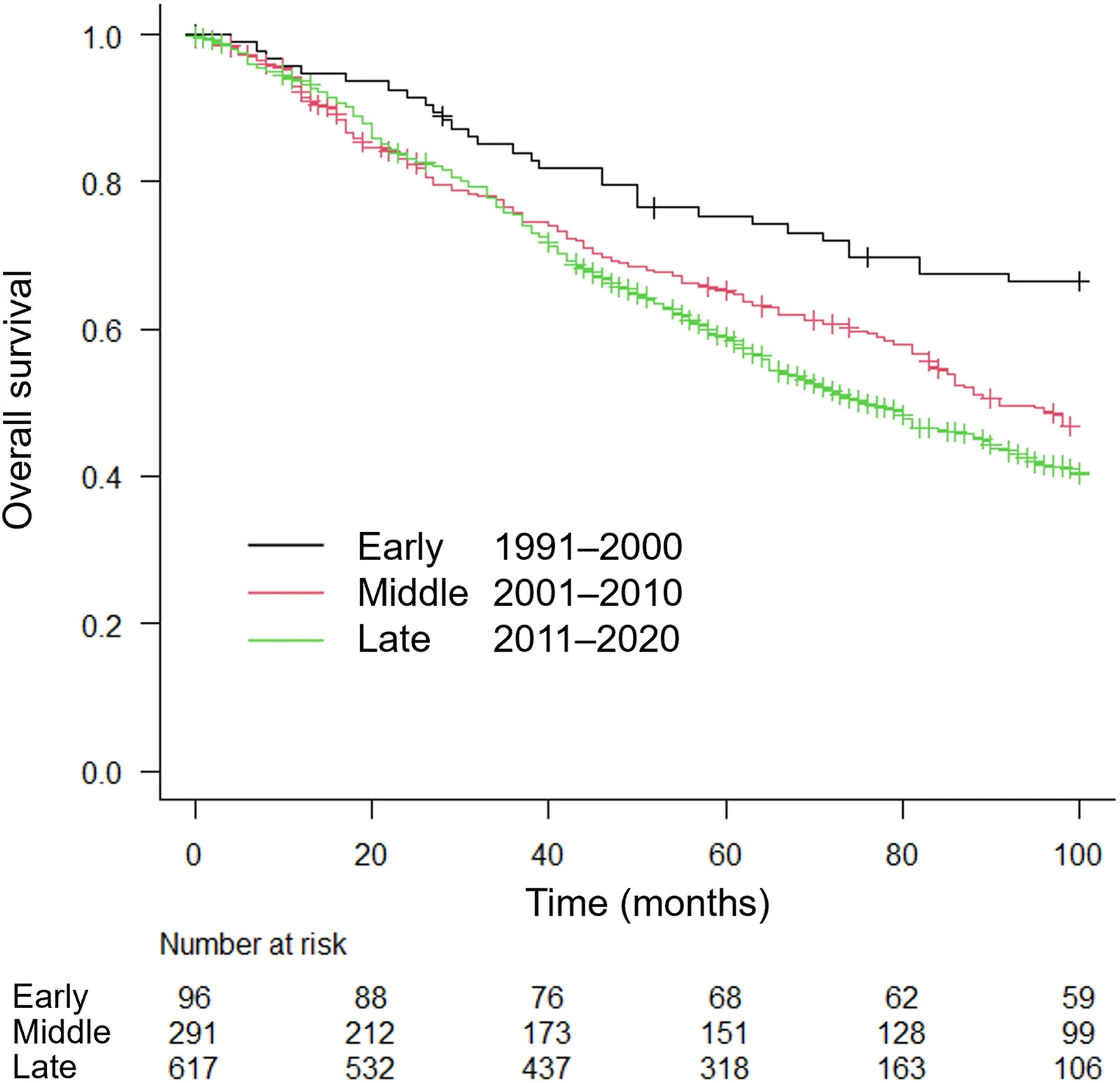
Kaplan-Meier overall survival curves according to diagnostic period. Five-year overall survival was 75.3%, 65.0%, and 58.7% in the early, middle, and late periods, respectively (log-rank *P* < .001).

**Fig 2:**
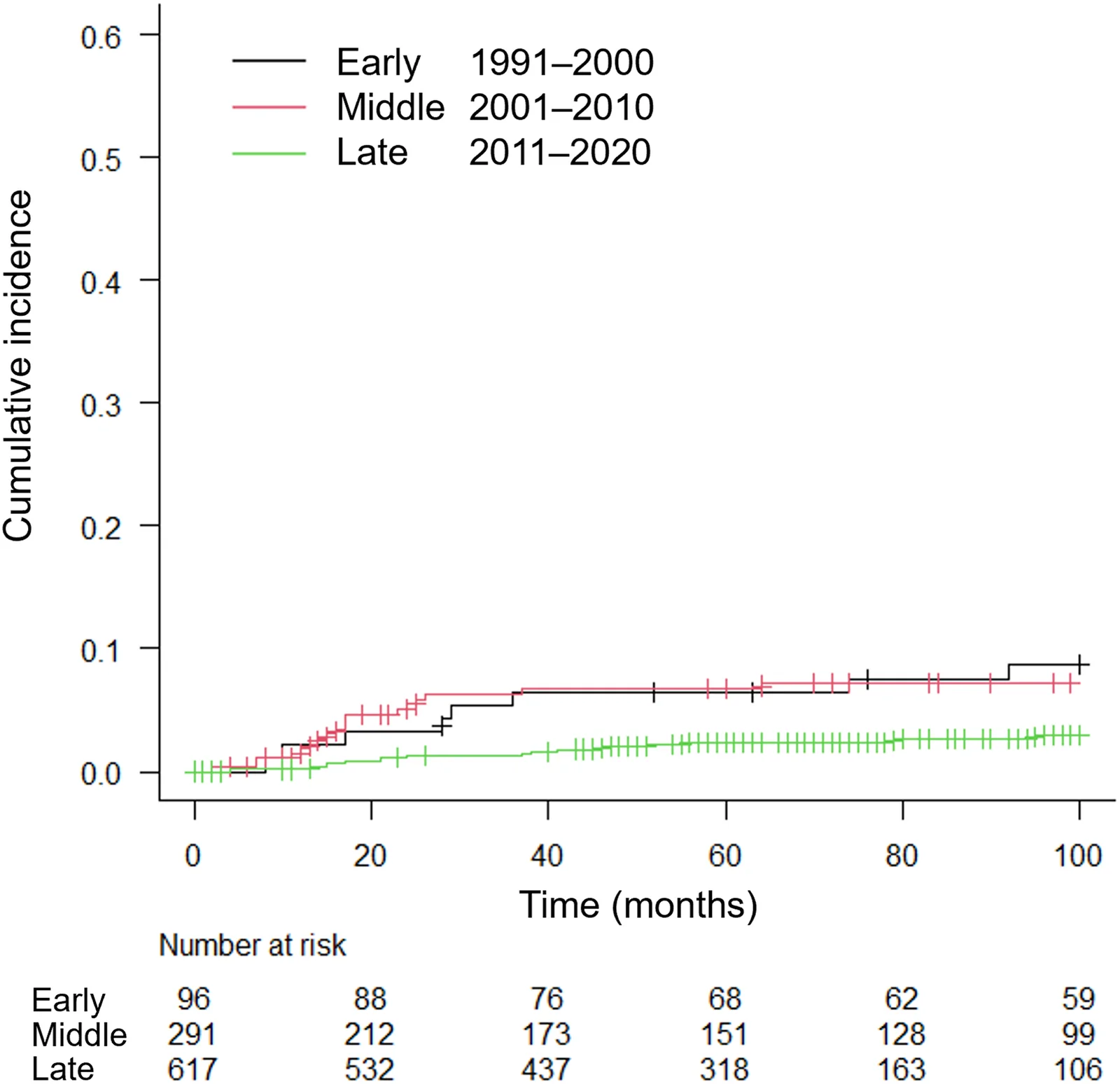
Cumulative incidence of cSCC-specific death according to diagnostic period. Five-year cumulative incidence was 6.4%, 6.7%, and 2.3% in the early, middle, and late periods, respectively (Gray test, *P* = .001).

In this 30-year cohort, overall survival worsened over time, whereas cSCC-specific mortality decreased in the most recent decade. Overall survival alone may obscure disease-specific outcomes because of competing mortality.^2^ The lower risk of cSCC-specific death persisted after adjustment for stage and other clinical variables, suggesting that stage migration alone does not fully explain the observed temporal trend. Patients became progressively older, tumors became smaller, and head and neck lesions increased, suggesting that the characteristics of diagnosed patients changed over time. These trends may reflect earlier detection and changes in case mix, although this retrospective study cannot determine their relative contributions. This finding should not be interpreted as evidence that improved care alone caused the observed temporal trend.

Limitations include the retrospective single-center design and the limited number of cSCC-specific deaths, which may have reduced model stability. Although cSCC-specific deaths were verified by medical record review, some misclassification of cause of death cannot be excluded.

These findings support the use of competing risk analysis in cSCC outcomes research, consistent with recent expert recommendations.^3^ This approach is particularly relevant in aging populations, where the burden of skin cancer continues to increase^4^, ^5^ and treatment decisions must balance cancer control against limited life expectancy and competing mortality risks.

### Declaration of generative AI and AI-assisted technologies in the writing process

During the preparation of this work, the authors used ChatGPT (OpenAI) to improve the clarity and readability of the text. After using this tool, the authors reviewed and edited the content as needed and take full responsibility for the content of the publication.

## Conflicts of interest

None disclosed.
